# Nurse-led telephone-based follow-up of secondary prevention after acute coronary syndrome: One-year results from the randomized controlled NAILED-ACS trial

**DOI:** 10.1371/journal.pone.0183963

**Published:** 2017-09-08

**Authors:** Daniel Huber, Robin Henriksson, Stina Jakobsson, Thomas Mooe

**Affiliations:** Department of Public Health and Clinical Medicine, Unit of Research, Education and Development—Östersund, Umeå University, Umeå, Sweden; Kurume University School of Medicine, JAPAN

## Abstract

**Background:**

Secondary prevention after acute coronary syndrome (ACS) could reduce morbidity and mortality, but guideline targets are seldom reached. We hypothesized that nurse-led telephone-based intervention would increase adherence.

**Methods:**

The NAILED ACS trial is a prospective, controlled, randomized trial. Patients admitted for ACS at Östersund hospital, Sweden, were randomized to usual follow-up by a general practitioner or a nurse-led intervention. The intervention comprised telephone follow-up after 1 month and then yearly with lifestyle counselling and titration of medications until reaching target values for LDL-C (<2.5 mmol/L) and blood pressure (BP; <140/90 mmHg) or set targets were deemed unachievable. This is a 12-month exploratory analysis of the intervention.

**Results:**

A total of 768 patients (396 intervention, 372 control) completed the 12-month follow-up. After titration at the 1-month follow-up, mean LDL-C was 0.38 mmol/L (95% CI 0.28 to 0.48, p<0.05), mean systolic BP 7 mmHg (95% CI 4.5 to 9.2, p<0.05), and mean diastolic BP 4 mmHg (95% CI 2.4 to 4.1, p<0.05) lower in the intervention group. Target values for LDL-C and systolic BP were met by 94.1% and 91.9% of intervention patients and 68.4% and 65.6% of controls (p<0.05). At 12 months, mean LDL was 0.3 mmol/L (95% CI 0.1 to 0.4, p <0.05), systolic BP 1.5 mmHg (95% CI -1.0 to 4.1, p = 0.24), and mean diastolic BP 2.1 mmHg (95% CI 0.6 to 3.6, p <0.05) lower in the intervention group. Target values for LDL-C and systolic BP were met in 77.7% and 68.9% of intervention patients and 63.2% and 63.7% of controls (p<0.05 and p = 0.125).

**Conclusion:**

Nurse-led telephone-based secondary prevention was significantly more efficient at improving LDL-C and diastolic BP levels than usual care. The effect of the intervention declined between 1 and 12 months. Further evaluation of the persistence to the intervention is needed.

## Introduction

Even though morbidity and mortality after acute coronary syndrome (ACS) have improved significantly over the last few decades, cardiovascular disease (CVD) is still the most prominent cause of premature death worldwide. Consequently, the World Health Organisation (WHO) issued a goal to reduce CVD deaths by 25% by 2025 [1]. Great advances have been achieved in the acute care of ACS, but a large part of the affliction in CVD is due to inadequate risk factor control to reduce future events [2]. There is vast and relatively undisputed knowledge that life-style interactions and effective, cheap, and readily available medications could improve long-term outcomes. Evidence-based guidelines for secondary preventive measures have been issued both nationally and internationally, and gradual improvements have been noted in the adherence to prescribed recommended medications in-hospital. However, studies of the achievement of guideline targets after the transition from in-hospital to outpatient care have shown unsatisfactory fulfilment, despite a high prevalence of recommended medications [3], [4]. New methods for outpatient secondary preventive follow-up have been called for, and the involvement of healthcare staff other than physicians, such as nurses and pharmacists, as well as new regimens of follow-up are recommended in the latest guidelines [5]. In small studies, telemedical methods have had interesting results in improving both adherence to medication and risk factors in the short-term [6, 7]. A more effective secondary preventive strategy must be both cost effective and easily implemented in today’s health care systems, with applicability to a large part of the target population and flexibility to meet each individual´s needs.

The primary aim of this study was to evaluate the 12-month outcomes for low-density lipoprotein cholesterol (LDL-C), systolic blood pressure (SBP), and diastolic blood pressure (DBP) in the Nurse-based Age-independent Intervention to Limit Evolution of Disease after Acute Coronary Syndrome (NAILED ACS) trial.

## Methods

### Design

The design of the NAILED-ACS trial was described previously [8]. Briefly, the trial is an ongoing single-centre, open, randomized, controlled population-based trial to evaluate nurse-led, telephone-based secondary prevention after ACS.

#### Trial participants

All patients admitted for ACS at Östersund County Hospital in Jämtland, Sweden, were eligible for inclusion. The hospital is the only secondary care provider in a large rural catchment area with a population of roughly 126 000. In the present study, patients admitted between 1 January 2010 and 31 December 2013 were included, and the last 12 months follow-up was conducted on 27 January 2015. We defined ACS as acute myocardial infarction (AMI) type 1 according to the universal definition of AMI, or unstable angina (UA) with electrocardiography (ECG) changes suggesting ischaemia [9]. Exclusion criteria were limited to an inability to adhere to the telephone follow-up due to deafness, aphasia, dementia, or other severe disease, non-Swedish or English speaking, and participation in another trial. An initial 3-month review of the study protocol did not reveal any missed cases outside the routine.

#### Recruitment and randomization

After obtaining written informed consent, we randomized patients into either an *intervention* group that was followed up by a nurse, or a *control* group followed up as standard care by a general practitioner (GP). The randomization was 1:1 using a computer-generated allocation sequence in blocks of four and stratified for sex and type of ACS. Further details of the randomization are described in the study protocol. [8]

#### Data collection

We collected baseline data at the in-hospital inclusion in an interview and by studying medical records. The data included co-morbidities, prevalent cardiovascular risk factors, prior cardiovascular events, and present medications. Blood pressure was measured in a seated position after 5 minutes of rest at baseline (1 month after discharge) and at follow-up by health care professionals at the patient´s closest health care facility and reported to the study nurses. Blood samples for lipid measurements were collected in the same manner. After the above measurements were made, both groups were contacted via telephone by a study nurse at baseline and for follow-up at 12, 24, and 36 months after discharge to assess smoking, current diet, exercise habits, and adherence to present medication. To detect a clinically relevant difference in SBP of 5 mmHg (standard deviation (SD) 19) and LDL-C of 0.5 mmol/L (SD 1.0) we calculated a sample size of at least 200 in each group (t-test with two independent samples, power 80%, two-tailed alpha 0.05). We included significantly more patients to maintain statistical power for further analyses after long-term follow-up (considering drop-outs i.e. because of deaths).

### Intervention and follow-up

#### Intervention

At each telephone contact, the study nurse advised the patient on life-style risk factors regarding diet, exercise, and smoking cessation. If needed according to the blood pressure and blood lipid measurements, the nurse and a joint study physician made personalized medical adjustments, which were then reassessed after 4 weeks. We repeated this routine until set secondary preventive targets were reached or deemed impossible to achieve.

#### Control

The study nurses contacted the control group for risk factor screening after blood pressure and blood sample measurements at the same time interval. We then forwarded the test results to the patient’s GP. No consultations or medical titrations were made with the telephone contact.

### Outcomes

As the present study had exploratory aims, our primary outcome was the mean difference in LDL-C levels at the 12-month follow-up between intervention and control patients. The secondary outcome was the mean difference in seated SBP and seated DBP. Targets in the study were set to LDL-C <2.5 mmol/L, SBP <140 mmHg, and DBP <90 mmHg in accordance with local guidelines. We also present the proportion reaching the target values for LDL-C, SBP, and DBP in both groups. To explore the effect of the titration, we examined the change in LDL-C, SBP, and DBP for patients with levels above target values at baseline, as well as the effect of the initial titration at 1 month by comparing levels to baseline. We also present the proportions on secondary preventive medication.

As of 23 March 2013, the target value for LDL-C in diabetics was changed to <1.8 mmol/L in the regional guidelines. We adopted this target in the intervention group to conform with guidelines, but for statistical reasons we use the initial target value in this study. The effect of this change in the target goal was published previously [10].

The primary outcome of the NAILED-ACS trial is LDL-C levels 36 months after discharge.

### Statistical analysis

We conducted all analyses according to the intention-to-treat principle, in which patients not adhering to the treatment plan are included but those lost to follow-up are excluded. The results are presented as means for continuous variables and percentages for categorical variables. We compared baseline characteristics and outcomes between the intervention and control groups using the two-sided independent sample t-test for continuous variables and chi-square test for categorical variables. As no further measurements were performed between 1 and 12 months in controls, we compared the results for intervention patients at 1 month after titrations and the baseline values of the controls. To explore the effect of medical titration, we performed a post hoc analysis of patients with values above set targets at baseline. In order to exclude potential interaction effects of the randomization variables (sex and type of ACS), we conducted a two-way ANOVA for all endpoints (LDL-C, SBP and DBP). A significance level of p <0.05 was used. All analyses were performed using IBM SPSS v22.0 software.

### Ethics

The study received ethical approval from the Regional Ethics Committee, Umeå, Sweden on October 28^th^, 2009

### Trial registration

Trial number: International Standard Randomized Controlled Trial Number (ISRCTN): 96595458. We completed this registration after the first inclusion, before the strict requirement of prospective registration of the ICMJE came to our attention. The study classifies therefor as retrospectively registered. The authors confirm that all on-going and related trials for this intervention are registered.

## Results

**Fig 1:**
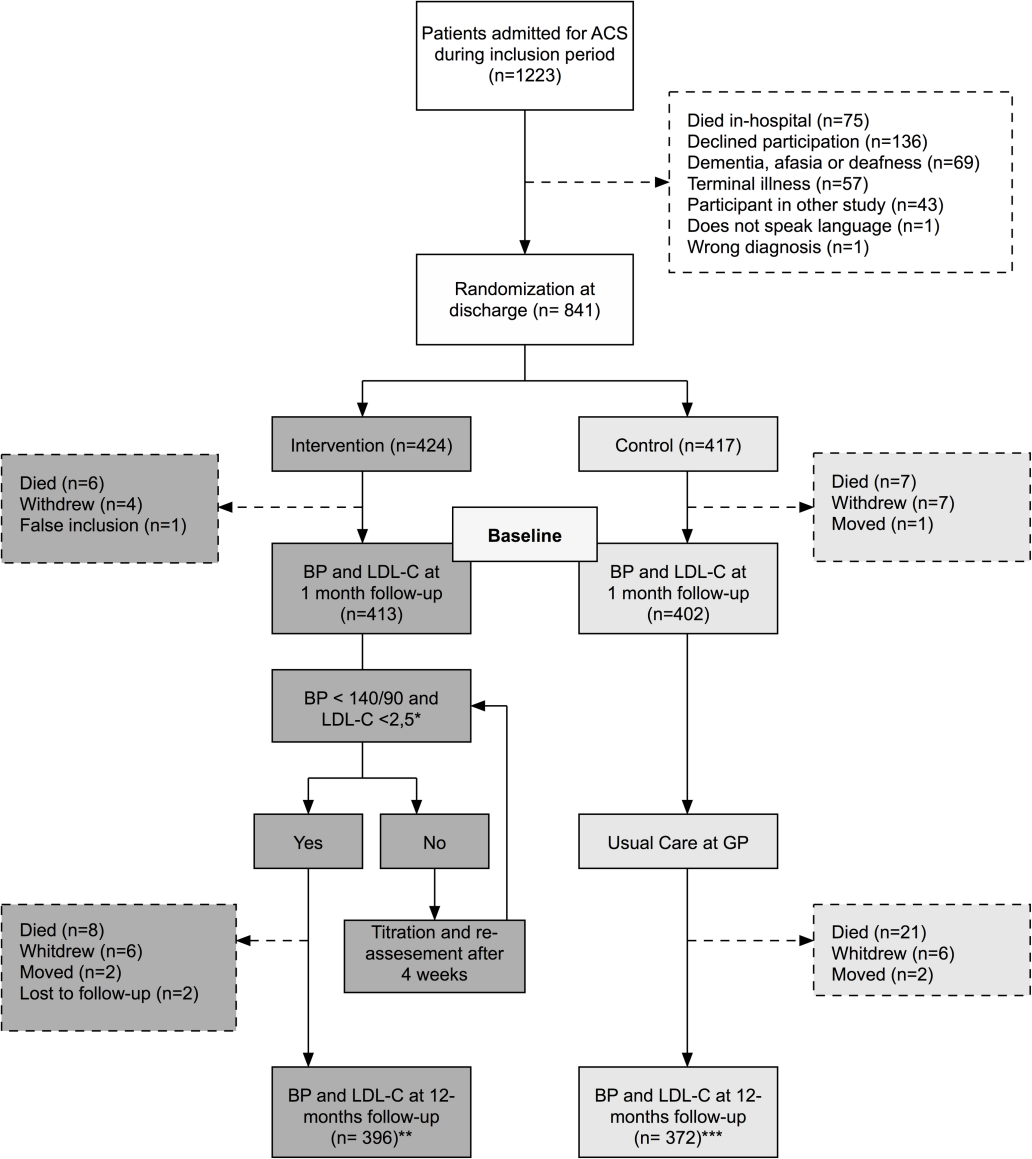
Flow chart. * as of 31 march 2013 target value for LDL-C in diabetic subjects is <1.8mmol/L, ** one patient declined at baseline, but re-entered at 12 months. *** one patient did only submit LDL-C but not BP.

Fig 1 presents the study profile during the first year. During the inclusion period, 1223 patients were admitted with ACS and screened; 75 died in-hospital and 307 were excluded. A total of 841 patients were included at discharge for the qualifying event, 768 of which completed the 12-month follow-up and were included in this study (396 intervention (51.6%), 372 control (48.4%)).

Only body mass index (BMI) differed statistically significant at baseline, which we deemed clinically insignificant in further analyses. Table 1 presents the baseline characteristics of the intervention and control groups. Table 2 presents the initial assessment at 1 month, before and after titration, as well as the 12-month follow-up. We found no significant interaction effects of sex or type of ACS on LDL-C level, SBP, or DBP.

**Table 1:** Patient characteristics by treatment group.

	Intervention (N = 396)		Control (N = 372)	p
Mean age, years (SD)	68.8 (11.7)		69.0 (11.4)	0.20
Male	273 (68.9%)		258 (69.4%)	0.90
Event at inclusion				
Unstable angina pectoris	26 (6.6%)		37 (9.9%)	0.09
NSTEMI	250 (63.1%)		230 (61.8%)	0.71
STEMI	120 (30.3%)		105 (28.2%)	0.53
At discharge for the qualifying event				
Body mass index, kg/m^2^ mean (SD)	27.7 (4.5)		27.0 (4.4)	0.03
eGFR, mL/min/1.73 m^2^ mean (SD)		78.7 (21.3)	78.9 (19.9)	0.90
mRS < 3	386 (97.5%)		364 (97.8%)	0.16
Diabetes	83 (21.0%)		68 (18.3%)	0.35
Atrial fibrillation	37 (9.6%)		27 (7.4%)	0.34
Congestive heart failure	2 (0.5%)		5 (1.3%)	0.13
Previous stroke	29 (7.3%)		25 (6.7%)	0.74
Previous myocardial infarction	65 (16.4%)		66 (17.7%)	0.62
Previous or current smoking	258 (65.2%)		224 (60.2%)	0.21

Data are given as n (%) unless otherwise noted. SD, standard deviation; NSTEMI, non-ST segment elevation myocardial infarction; STEMI, ST segment elevation myocardial infarction; eGFR, estimated glomerular filtration rate, calculated by the CKD-EPI formula; mRS, modified Rankin score

**Table 2:** Results at baseline (1 month) before and after titration and at 1 year.

	Baseline - before titration			Baseline - after titration			12 months - before titration		
	Intervention	Control	p	Intervention	Control	p	Intervention	Control	p
SBP, mmHg; mean (SD)	131.5 (19.0)	131.8 (18.9)	0.85	124.9 (13.3)	131.8 (18.9)	<0.001	131.5 (17.1)	133.0 (19.2)	0.24
DBP, mmHg; mean (SD)	76.9 (10.6)	77.7 (11.2)	0.33	73.7 (9.5)	77.7 (11.2)	<0.001	76.0 (10.2)	78.2 (11.3)	0.007
LDL-C, mmol/L; mean (SD)	2.16 (0.75)	2.25 (0.87)	0.11	1.9 (0.46)	2.3 (0.87)	<0.001	2.1 (0.74)	2.4 (0.96)	<0.001
SBP/DBP < 140/90	251 (63.5)	255 (60.5)	0.38	353 (89.4)	225 (60.5)	<0.001	257 (64.9%)	218 (58.6%)	0.07
LDL-C < 2.5 mmol/L	268 (68.7)	251 (68.4)	0.82	369 (94.1)	251 (68.4)	<0.001	289 (77.7%)	230 (63.2%)	<0.001
Antilipids	365 (92.2%)	336 (90.3%)	0.37				367 (92.7%)	329 (88.4%)	0.04
Beta-blockers	359 (90.7%)	338 (90.9%)	0.92				351 (88.6%)	329 (88.4%)	0.93
Alpha-blockers	10 (2.5%)	9 (2.4%)	0.92				8 (2.0%)	14 (3.8%)	0.14
ACE inhibitors	232 (58.6%)	230 (61.8%)	0.36				209 (52.8%)	196 (52.7%)	0.98
ARBs	85 (21.5%)	67 (18.0%)	0.48				116 (29.3%)	88 (23.7%)	0.13

Crude p-values. Data are presented as n (%) unless otherwise noted. SD, standard deviation; SBP, systolic blood pressure; DBP, diastolic blood pressure; LDL-C Low density lipoprotein cholesterol; ACE, angiotensin converting enzyme; ARB, angiotensin II receptor blocker.

**Fig 2:**
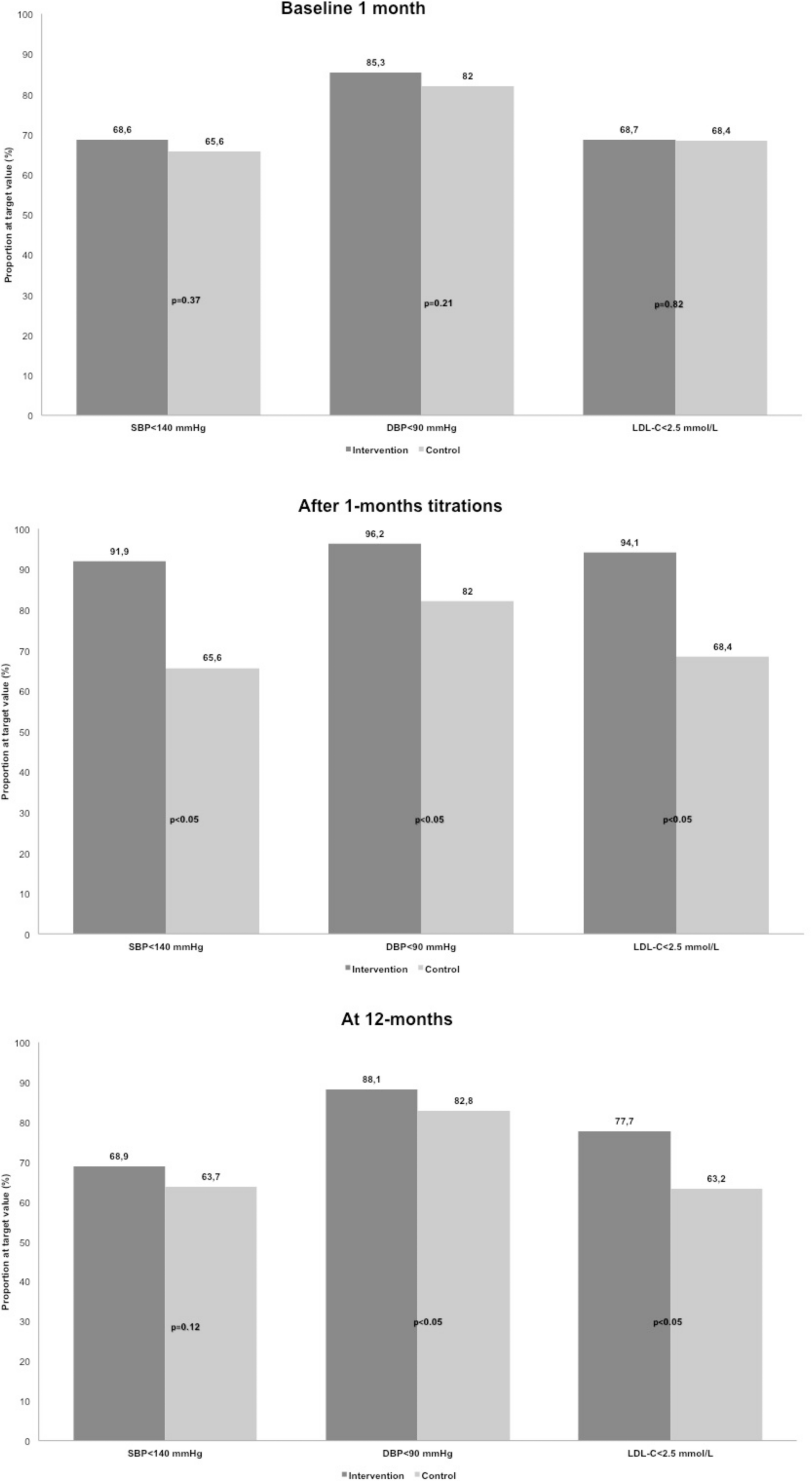
Proportion at target value. SBP, systolic blood pressure; DBP diastolic blood pressure; LDL-C, Low-Density Lipoprotein Cholesterol.

The proportion of participants reaching set target values at each control is presented in Fig 2.

### LDL-C

After initial titration, LDL-C was 0.38 mmol/L lower (95% CI 0.28 to 0.48, p<0.001) in the intervention group than the control group at baseline (Table 2). At the 12-month follow-up, the mean difference was 0.27 mmol/L (95% CI 0.15 to 0.39, p <0.001). In patients with LDL-C levels above the target value at 1 month (baseline), the mean difference at 12 months was 0.40 mmol/L (95% CI 0.18–0.62, p = 0.001), with a lower LDL-C level in the intervention group compared to controls.

### Blood pressure

At baseline, there were no differences in mean SBP or DBP between patients in the intervention and control groups (Table 2). After initial titration in the intervention group, the mean SBP was 6.9 mmHg lower (95% CI 4.5 to 9.2, p<0.001) and mean DBP 4.0 mmHg lower (95% CI 2.5 to 5.5, p<0.001) than in the control group. At the 12-month follow-up, mean SBP was 1.5 mmHg lower (95% CI -1.0 to 4.1, p = 0.24) and DBP 2 mmHg lower (95% CI 0.6 to 3.6, p = 0.007) in the intervention group compared to controls. In patients with blood pressure measurements above target values at 1 month (baseline), mean SBP was 2 mmHg lower (95% CI -2.6 to 6.8, p = 0.38) and mean DBP 7 mmHg lower (95% CI 3.7 to 10.0, p <0.001) in the intervention group.

### Medication

As seen in Table 2, a difference in present medication was only found between the groups at 12 months for lipid-lowering therapy.

## Discussion

The majority of patients included in this relatively unselected cohort were available for a 12-month follow-up (91.3%, n = 768). Implementation, with rates and reasons for non-participation, was published previously [11]. In the group comparison at 12 months, significantly lower LDL-C levels and DBP were measured, with a larger proportion of intervention patients reaching the set targets compared to the control group. A trend towards reduced SBP compared to the control group was also seen, though the difference was not significant. In patients with levels above target values at baseline and in need of adjusted medical treatment, the differences were more pronounced, but still insignificant for SBP.

In contrast to the findings here, the EUROASPIRE IV study found that only two-fifths reached a blood pressure <140/90 mmHg (Sweden 62.4%) and less than two-thirds LDL-C <2.5 mmol/L (Sweden 65.9%) [3]. Jorstad et al investigated an intensified multifaceted nurse-led secondary prevention for 6 months in the RESPONSE study with the Framingham risk score as the main outcome. After the intensified follow-up period, 72% of intervention patients achieved a SBP <140 mmHg, 87% a DBP <90 mmHg, and 80% LDL-C <2.5 mmol/L [12]. The EUROASPIRE IV study showed that, despite large regional differences, the overall use of recommended secondary preventive drugs was high (lipid-lowering drugs 86.6%, four-fifths on beta-blockers and three-quarters on ACE inhibitors or ARBs), which seems to concur with other studies, as well as the Swedish registry for cardiovascular care (SWEDHEART) [13]. In the present study, the use of recommended drugs was at the same level, and differed significantly only in regards to lipid-lowering medication at 12 months.

So are these findings clinically relevant? In a meta-analysis, statin-induced reduction in LDL-C reduced morbidity and mortality in cardiovascular disease in a linear manner irrespective of base line value. [14] A meta-analysis on prevention of cardiovascular disease by blood pressure lowering drugs showed similar results with a linear reduction of morbidity and mortality irrespective of pre-treatment values. [15] There is an on-going debate on what the optimal targets are for lipids and BP in secondary prevention, but guidelines still stipulate that any reductions in LDL-C and BP in secondary prevention are mortality reducing in both the individual and society. [16]

Kotseva et al concluded that a high prevalence of recommended drugs does not equal adequate risk factor control, probably due to an initial low-dose regimen and lack of titration. Redón et al identified primary care setting, risk factor values close to normal, and multiple health contacts as explanations for the lack of titration [17]. Our study was designed to try to reduce therapeutic inertia, the health care provider’s inability to act on identified risk factors [18], by providing nurse-led follow-up with strict instructions not to accept risk factor values outside set targets. Our study confirms the conclusion of Kotseva et al and clearly indicates that it is possible for the vast majority of patients, even in an unselected cohort, to reach set secondary preventive targets via individualized titration of medication with strict adherence to evidence-based target values.

The question of why the effect of the intervention is so poorly maintained over 12 months is important to answer and will be analysed in another study. Most intervention studies of secondary prevention are short and lack data from 12 months and beyond. In a recent meta-analysis, Snaterse et al found that nurse-controlled secondary prevention for CVD was effective at lowering SBP and LDL-C levels if combined with medical titration in a high intensity strategy (i.e., multiple health care contacts at short intervals) [19]. In the RESPONSE study with four interventions during the first 6 months, a larger proportion of intervention patients were on target for SBP and LDL-C, but not DBP. They did not see any significant decrease in SBP or LDL-C in the intervention group after 12 months [12]. In the SPRINT study, excluding diabetics and patients with a history of stroke, the reduction in blood pressure (to an even greater extent than in our study) was maintained for a mean of 3.26 years. Both cardiovascular morbidity and mortality decreased, with more adverse events in the intensified treatment group but no contradictory side effects in any age group [20]. The SPRINT intervention included visits every 3 months, and similarly short intervals between health care contacts may be necessary to maintain a high level of adherence.

Studies of adherence are of varying quality and poor generalizability [21], but the patient´s own incentive for medication and low barriers to health care contact have been stipulated to increase adherence [22]. In the NAILED intervention patients had the ability to contact the study nurses at will, but no contacts were planned between the final titration at baseline and the 12-month follow-up. A more persistent effect of the intervention may be seen with more frequent contacts during the first year. This aspect was considered as we planned our study, but we concluded that more visits would make the methodology too costly and difficult to implement in many clinical practices. The outcomes of the main NAILED-ACS trial will reveal whether the effect of the intervention increases with persistent follow-up over the planned 36 months.

### Limitations and strengths

The aim of this study was to test a pragmatic secondary preventive programme in a population-based, relatively unselected patient cohort, which should well represent the clinical situation in most practices. Furthermore, the study design does not require frequent travel to hospital outpatient clinics, which increases the adaptability to many clinical settings. This real-world situation comes at the cost of an absence of more pre-defined exclusion criteria, which could be argued to reduce the method’s reproducibility. Another possible limitation would be the single-centre design, which may limit the applicability of the results in other clinical settings.

Randomized controlled trials are preferably blinded to both participants and study professionals. Our study is of an open design, as blinding would be both practically and ethically impossible. Participation and documentation of treatment were recorded in each patient’s medical record, which was disclosed to all with access. By design we performed risk factor measurements in all participants and forwarded the test results to the control group’s GP. We cannot exclude this leading to the control group being subjected to a more active follow-up than would otherwise have occurred due to the GP´s own incentive or patients’ heightened awareness. However, this most likely led to an underestimation, rather than exaggeration, of the effect of the intervention. There is also a potential risk that a GP who found out that their patient participated in a clinical trial may not have intervened according to guidelines in order to not interfere, being unaware of the study design or whether the patient belonged to the intervention or control group. This would have led to an overestimation of the intervention. Finally, the low exclusion and dropout rates led to an increase in the external validity of the study.

### Conclusion

Nurse-led telephone-based follow-up of a secondary prevention programme was highly applicable to a standard clinical setting and led to significantly lower LDL-C and DBP after 12 months compared to usual care. A larger proportion of the intervention group also reached target values for LDL-C and DBP at 12 months compared to baseline. The effect of the intervention was more pronounced after initial titration but declined during the first 12 months. The long-term effect of the intervention remains to be evaluated, and also factors contributing to persistence to the intervention.

## Supporting information

S1 Table CONSORT checklist. (DOC)

S1 Text Study protocol. (DOC)

## References

[1] Smith SC Jr., Collins A, Ferrari R, Holmes DR Jr., Logstrup S, McGhie DV, et al Our time: a call to save preventable death from cardiovascular disease (heart disease and stroke). Eur Heart J. 2012;33(23):2910–6. doi: 10.1093/eurheartj/ehs313 . 22988314 10.1093/eurheartj/ehs313

[2] Bjorck L, Rosengren A, Bennett K, Lappas G, Capewell S. Modelling the decreasing coronary heart disease mortality in Sweden between 1986 and 2002. Eur Heart J. 2009;30(9):1046–56. doi: 10.1093/eurheartj/ehn554 . 19141562 10.1093/eurheartj/ehn554

[3] Kotseva K, Wood D, De Bacquer D, De Backer G, Ryden L, Jennings C, et al EUROASPIRE IV: A European Society of Cardiology survey on the lifestyle, risk factor and therapeutic management of coronary patients from 24 European countries. Eur J Prev Cardiol. 2016;23(6):636–48. doi: 10.1177/2047487315569401 . 25687109 10.1177/2047487315569401

[4] Chen HY, Saczynski JS, Lapane KL, Kiefe CI, Goldberg RJ. Adherence to evidence-based secondary prevention pharmacotherapy in patients after an acute coronary syndrome: A systematic review. Heart Lung. 2015;44(4):299–308. doi: 10.1016/j.hrtlng.2015.02.004 . 25766041 10.1016/j.hrtlng.2015.02.004 PMC8075173

[5] Perk J, Hambraeus K, Burell G, Carlsson R, Johansson P, Lisspers J. Study of Patient Information after percutaneous Coronary Intervention (SPICI): should prevention programmes become more effective? EuroIntervention. 2015;10(11):e1–7. doi: 10.4244/EIJV10I11A223 . 24472705 10.4244/EIJV10I11A223

[6] Kotb A, Hsieh S, Wells GA. The effect of telephone support interventions on coronary artery disease (CAD) patient outcomes during cardiac rehabilitation: a systematic review and meta-analysis. PLoS One. 2014;9(5):e96581 doi: 10.1371/journal.pone.0096581 ; PubMed Central PMCID: PMCPMC4010507. 24798429 10.1371/journal.pone.0096581 PMC4010507

[7] Neubeck L, Redfern J, Fernandez R, Briffa T, Bauman A, Freedman SB. Telehealth interventions for the secondary prevention of coronary heart disease: a systematic review. Eur J Cardiovasc Prev Rehabil. 2009;16(3):281–9. doi: 10.1097/HJR.0b013e32832a4e7a . 19407659 10.1097/HJR.0b013e32832a4e7a

[8] Mooe T, Bjorklund F, Graipe A, Huber D, Jakobsson S, Kajermo U, et al The Nurse-Based Age Independent Intervention to Limit Evolution of Disease After Acute Coronary Syndrome (NAILED ACS) Risk Factor Trial: Protocol for a Randomized Controlled Trial. JMIR Res Protoc. 2014;3(3):e42 doi: 10.2196/resprot.3466 ; PubMed Central PMCID: PMCPMC4147706. 25131960 10.2196/resprot.3466 PMC4147706

[9] Thygesen K, Alpert JS, White HD, Joint ESCAAHAWHFTFftRoMI. Universal definition of myocardial infarction. J Am Coll Cardiol. 2007;50(22):2173–95. doi: 10.1016/j.jacc.2007.09.011 . 18036459 10.1016/j.jacc.2007.09.011

[10] Jakobsson S, Huber D, Bjorklund F, Mooe T. Implementation of a new guideline in cardiovascular secondary preventive care: subanalysis of a randomized controlled trial. BMC Cardiovasc Disord. 2016;16:77 doi: 10.1186/s12872-016-0252-0 ; PubMed Central PMCID: PMCPMC4851797. 27129980 10.1186/s12872-016-0252-0 PMC4851797

[11] Huber D, Henriksson R, Jakobsson S, Stenfors N, Mooe T. Implementation of a telephone-based secondary preventive intervention after acute coronary syndrome (ACS): participation rate, reasons for non-participation and 1-year survival. Trials. 2016;17(1):85 doi: 10.1186/s13063-016-1203-x ; PubMed Central PMCID: PMCPMC4753651. 26876722 10.1186/s13063-016-1203-x PMC4753651

[12] Jorstad HT, von Birgelen C, Alings AM, Liem A, van Dantzig JM, Jaarsma W, et al Effect of a nurse-coordinated prevention programme on cardiovascular risk after an acute coronary syndrome: main results of the RESPONSE randomised trial. Heart. 2013;99(19):1421–30. doi: 10.1136/heartjnl-2013-303989 ; PubMed Central PMCID: PMCPMC3786610. 23813851 10.1136/heartjnl-2013-303989 PMC3786610

[13] SWEDEHEART Annual report 2015 [Internet]. http://www.ucr.uu.se/swedeheart/index.php/dokument-sh/arsrapporter; 2016 [cited 27 May 2016]. Available from: http://www.ucr.uu.se/swedeheart/index.php/dokument-sh/arsrapporter

[14] Cholesterol Treatment Trialists C, Mihaylova B, Emberson J, Blackwell L, Keech A, Simes J, et al The effects of lowering LDL cholesterol with statin therapy in people at low risk of vascular disease: meta-analysis of individual data from 27 randomised trials. Lancet. 2012;380(9841):581–90. doi: 10.1016/S0140-6736(12)60367-5 ; PubMed Central PMCID: PMCPMC3437972. 22607822 10.1016/S0140-6736(12)60367-5 PMC3437972

[15] Law MR, Morris JK, Wald NJ. Use of blood pressure lowering drugs in the prevention of cardiovascular disease: meta-analysis of 147 randomised trials in the context of expectations from prospective epidemiological studies. BMJ. 2009;338:b1665 doi: 10.1136/bmj.b1665 ; PubMed Central PMCID: PMCPMC2684577. 19454737 10.1136/bmj.b1665 PMC2684577

[16] Authors/Task Force M, Piepoli MF, Hoes AW, Agewall S, Albus C, Brotons C, et al 2016 European Guidelines on cardiovascular disease prevention in clinical practice: The Sixth Joint Task Force of the European Society of Cardiology and Other Societies on Cardiovascular Disease Prevention in Clinical Practice (constituted by representatives of 10 societies and by invited experts): Developed with the special contribution of the European Association for Cardiovascular Prevention & Rehabilitation (EACPR). Eur J Prev Cardiol. 2016;23(11):NP1–NP96. doi: 10.1177/2047487316653709 . 27353126 10.1177/2047487316653709

[17] Redon J, Coca A, Lazaro P, Aguilar MD, Cabanas M, Gil N, et al Factors associated with therapeutic inertia in hypertension: validation of a predictive model. J Hypertens. 2010;28(8):1770–7. doi: 10.1097/HJH.0b013e32833b4953 . 20531224 10.1097/HJH.0b013e32833b4953

[18] Okonofua EC, Simpson KN, Jesri A, Rehman SU, Durkalski VL, Egan BM. Therapeutic inertia is an impediment to achieving the Healthy People 2010 blood pressure control goals. Hypertension. 2006;47(3):345–51. doi: 10.1161/01.HYP.0000200702.76436.4b . 16432045 10.1161/01.HYP.0000200702.76436.4b

[19] Snaterse M, Dobber J, Jepma P, Peters RJ, Ter Riet G, Boekholdt SM, et al Effective components of nurse-coordinated care to prevent recurrent coronary events: a systematic review and meta-analysis. Heart. 2016;102(1):50–6. doi: 10.1136/heartjnl-2015-308050 ; PubMed Central PMCID: PMCPMC4717438. 26567234 10.1136/heartjnl-2015-308050 PMC4717438

[20] Group SR, Wright JT Jr., Williamson JD, Whelton PK, Snyder JK, Sink KM, et al A Randomized Trial of Intensive versus Standard Blood-Pressure Control. N Engl J Med. 2015;373(22):2103–16. doi: 10.1056/NEJMoa1511939 ; PubMed Central PMCID: PMCPMC4689591. 26551272 10.1056/NEJMoa1511939 PMC4689591

[21] Nieuwlaat R, Wilczynski N, Navarro T, Hobson N, Jeffery R, Keepanasseril A, et al Interventions for enhancing medication adherence. Cochrane Database Syst Rev. 2014;11:CD000011 doi: 10.1002/14651858.CD000011.pub4 . 25412402 10.1002/14651858.CD000011.pub4 PMC7263418

[22] Osterberg L, Blaschke T. Adherence to medication. N Engl J Med. 2005;353(5):487–97. doi: 10.1056/NEJMra050100 . 16079372 10.1056/NEJMra050100

